# Comparative short- and long-term outcomes of TOETVA, ETGUA, and COT in thyroid cancer: a propensity score-matched study

**DOI:** 10.3389/fonc.2025.1606568

**Published:** 2025-10-08

**Authors:** Cong Bian, Zhenmeng Lin, Mingfang Yan, Shaokun Weng, Chao Xie, Wen Pan, Chenlan Huang, Guiren Fang

**Affiliations:** ^1^ Department of Head and Neck Surgery, Clinical Oncology School of Fujian Medical University & Fujian Cancer Hospital, Fuzhou, China; ^2^ Department of Anesthesiology, Clinical Oncology School of Fujian Medical University & Fujian Cancer Hospital, Fuzhou, China; ^3^ Department of Urological Oncology, Clinical Oncology School of Fujian Medical University & Fujian Cancer Hospital, Fuzhou, China

**Keywords:** thyroid cancer, transoral endoscopic thyroidectomy vestibular approach, gasless unilateral axillary endoscopic thyroidectomy, open thyroidectomy, wound satisfaction, quality of life

## Abstract

**Objective:**

This study aims to compare the short-term and long-term outcomes of three surgical approaches for thyroid cancer: Transoral Endoscopic Thyroidectomy Vestibular Approach (TOETVA), Gasless Unilateral Axillary Endoscopic Thyroidectomy (ETGUA), and Conventional Open Thyroidectomy (COT).

**Methods:**

A total of 466 thyroid cancer patients who underwent surgery were initially included. After propensity score matching (PSM), 318 patients were analyzed, with 106 patients in each group (TOETVA, ETGUA, and COT). The primary outcomes compared included surgical parameters (incision length, blood loss, operation time, lymph node dissection), postoperative inflammation (WBC, CRP, IL-6), postoperative complications, postoperative pain (Visual Analog Scale, VAS), scar assessment (Vancouver Scar Scale, VSS), wound satisfaction, costs, and quality of life (36-Item Short Form Health Survey, SF-36).

**Results:**

After matching, the operation time for TOETVA was longer than that for both ETGUA and COT. The number of lymph nodes dissected in ETGUA was fewer than in TOETVA and COT. There were no significant differences in postoperative complications, VAS scores, costs, or hospital stay among the three groups. On the first postoperative day, TOETVA and ETGUA showed higher levels of WBC and CRP than COT, but no significant differences were observed by day three. TOETVA had the shortest incision and the lowest VSS score. Wound satisfaction was significantly higher in both TOETVA and ETGUA compared to COT, with no significant difference between TOETVA and ETGUA. In terms of quality of life, some dimensions in TOETVA and ETGUA were higher than in COT. Each surgical approach has its own advantages and disadvantages.

**Conclusion:**

TOETVA demonstrated the shortest incision and the lowest VSS score, while both TOETVA and ETGUA showed the best wound satisfaction and specific domains of quality of life. However, TOETVA had the longest operation time, and ETGUA had the fewest lymph nodes dissected.

## Introduction

Thyroid cancer is one of the most common malignancies worldwide, with its incidence steadily rising over recent decades, particularly among women aged 20-29 years (1–3). As the number of younger patients increases, there is a growing emphasis on both medical and aesthetic outcomes in treatment. Fortunately, thyroid cancer generally has a good prognosis with a high survival rate, making quality of life an important consideration for both patients and healthcare providers (4, 5).

Conventional open thyroidectomy (COT) remains a key treatment approach for thyroid cancer. However, one of its major drawbacks is the visible surgical scar on the anterior neck, which can be particularly prominent in patients prone to developing hypertrophic scars (6). This can lead to significant cosmetic concerns and psychological distress (7). Consequently, improving the aesthetic outcome of surgical scars, ensuring effective tumor control, enhancing postoperative recovery, and optimizing the patient’s quality of life have become critical objectives in thyroid cancer surgery.

Laparoscopic surgery has garnered increasing attention due to its numerous advantages, such as magnification, which allows for a clearer surgical field and improved precision during surgery. Furthermore, laparoscopic approaches typically involve smaller, less noticeable incisions, providing excellent cosmetic benefits while ensuring effective treatment outcomes (8–10). As a result, laparoscopic thyroidectomy has become increasingly popular among both thyroid surgeons and patients. Various laparoscopic approaches have been developed, including the Transoral Endoscopic Thyroidectomy Vestibular Approach (TOETVA) and Gasless Unilateral Axillary Endoscopic Thyroidectomy (ETGUA) (11, 12). Each of these approaches has its own set of advantages and disadvantages, and there is still no consensus on which technique is the most suitable for clinical use. This study aims to compare the short-term and long-term outcomes of three surgical approaches for thyroid cancer: TOETVA, ETGUA, and COT.

## Methods

### Patients

**Figure 1:**
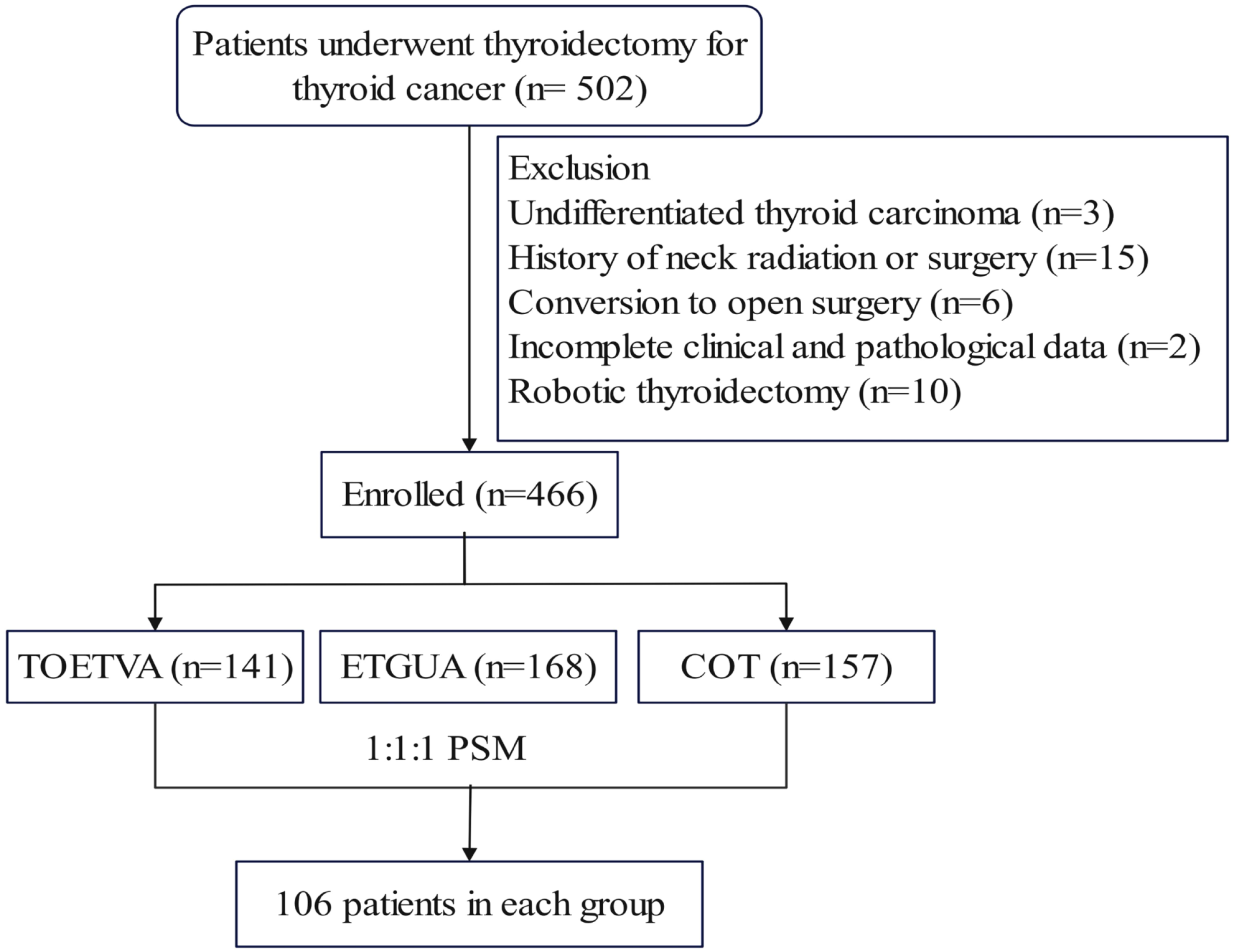
Flow diagram depicting patient selection. PSM, propensity score matching.

This prospective non-randomized interventional study included patients who underwent surgical treatment for thyroid cancer at Fujian Cancer Hospital from March 2022 to August 2023. The inclusion criteria were: (1) histopathologically confirmed differentiated thyroid cancer; (2) no distant organ metastasis on imaging studies; and (3) complete clinical and pathological data. The exclusion criteria were: (1) a history of neck radiation, previous neck surgery, or trauma; (2) Grade III hyperthyroidism; (3) conversion from endoscopic surgery to open surgery during the procedure; (4) robotic thyroidectomy, and (5) concurrent psychiatric disorders. As shown in the flowchart (**Figure 1**), a total of 318 thyroid cancer patients were included, with 106 patients in each group.

### Surgical technique

TOETVA: The patient is positioned in a supine position with hyperextension of the neck. A small horizontal incision of approximately 3-4 cm is made at the anterior vestibule of the oral cavity, just above the upper gingiva. The surgical site is prepared by injecting a solution containing adrenaline for tissue expansion in the anterior cervical subcutaneous space. This is followed by the application of CO2 insufflation to maintain the cervical space. The dissection is carried out in a subperiosteal plane, starting from the mandible and extending toward the upper margin of the sternum. The thyroid gland is exposed through this corridor, and thyroidectomy is performed.

ETGUA: The patient is placed in a supine position with the shoulders slightly elevated. A skin incision of approximately 8 cm is made along the natural axillary skin crease. The skin flap is dissected along the superficial fascia of the pectoralis major muscle. A specialized retractor is inserted into the axillary space between the sternal and clavicular heads of the sternocleidomastoid muscle. After identifying the omohyoid and hyoglossus muscles, a suspension retractor is placed to aid dissection. The thyroid gland is then exposed through this route, and thyroidectomy is performed.

**Figure 2:**
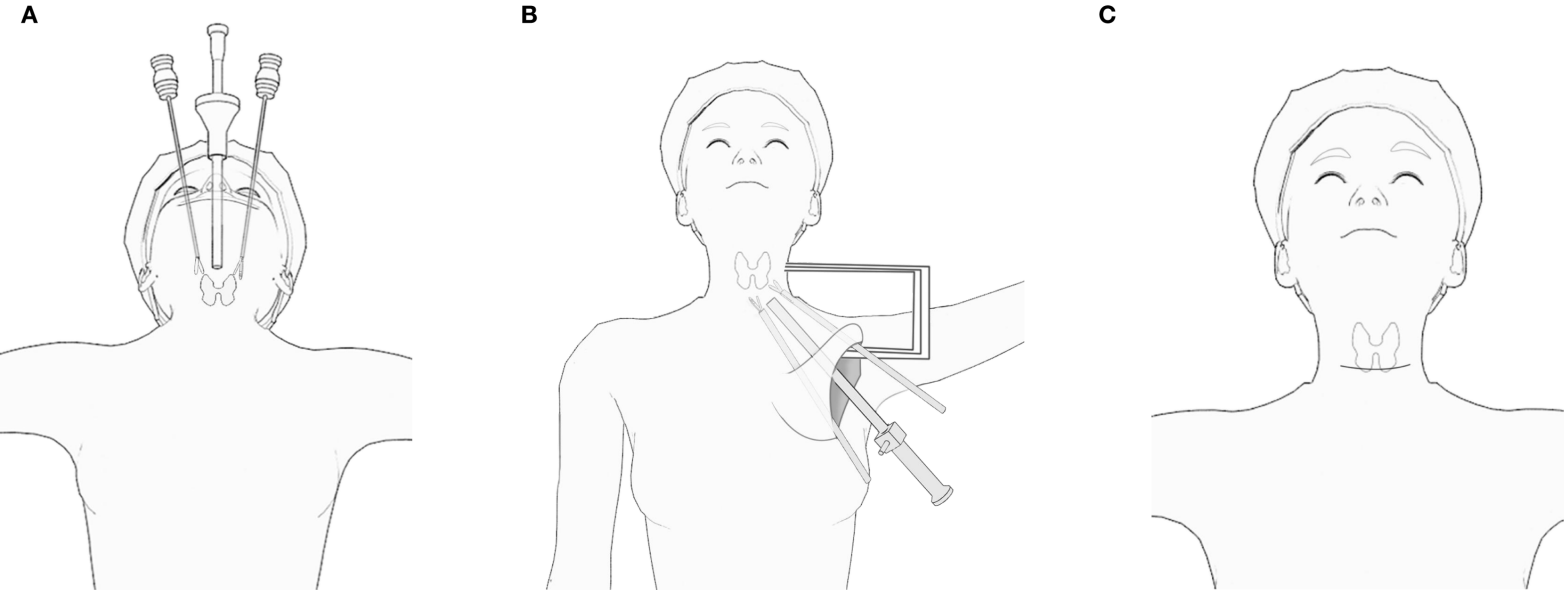
Surgical diagrams illustrating the procedures: **(A)** TOETVA; **(B)** ETGUA; **(C)** COT.

COT: A skin incision of approximately 8 cm is made in a transverse direction 1-2 cm above the sternal notch, following the natural skin lines. The skin flap is dissected subcutaneously, and the strap muscles are separated along the midline (cervical line) to expose the thyroid gland. Thyroidectomy is then performed after careful dissection of surrounding structures. **Figure 2** illustrates the surgical diagrams for these procedures.

### Data collection and outcomes

The preoperative, postoperative day (POD1) 1, and day 3 White blood cell (WBC), serum C-reactive protein (CRP), and Interleukin-6 (IL-6) levels were measured to assess the inflammatory response.

Nonsteroidal anti-inflammatory drugs (NSAIDs) were routinely used for pain relief on postoperative days 1 and 2. The pain intensity was assessed using the Visual Analog Scale (VAS) under the guidance of a professional nurse on the morning of postoperative days 1 to 3.

The drainage tube was removed when the drainage fluid became clear and the volume of drainage was less than 20 mL per day for TOETVA and COT, and less than 50 mL for ETGUA.

A parathyroid hormone (PTH) level < 12 pg/mL at 6 hours postoperatively is considered indicative of hypoparathyroidism. At 6 months of follow-up, a PTH level ≤ 12 pg/mL or the development of clinical symptoms of permanent hypocalcemia should be considered as permanent hypoparathyroidism (13, 14).

Recurrent laryngeal nerve injury is characterized by hoarseness, dysphagia, aphonia, or dyspnea postoperatively in patients without preoperative hoarseness. Preoperative laryngoscopy reveals normal vocal cords. Following surgery, patients may experience voice changes, coughing while swallowing, loss of voice, or difficulty breathing. Permanent injury is defined as damage lasting for more than 6 months (15, 16).

The Vancouver Scar Scale (VSS) was used to assess postoperative scarring. The VSS evaluates four parameters: height, pliability, vascularity, and pigmentation, with a score range of 0 to 15. A higher score indicates more severe scarring (17). The wound satisfaction score ranges from 0 to 10, with 0 indicating very unsatisfied and 10 indicating very satisfied (18).

This study used the 36-Item Short Form Health Survey (SF-36) to assess quality of life preoperatively and at 1, 3, 6, and 12 months postoperatively. The SF-36 includes 8 dimensions: physical functioning (PF), role limitations due to physical problems (RP), bodily pain (BP), general health perceptions (GH), vitality (VT), social functioning (SF), role limitations due to emotional problems (RE), and mental health (MH). Each dimension’s scores range from 0 to 100, with higher scores indicating better quality of life. This scale is widely used to evaluate the quality of life in thyroid cancer patients (19–21).

### Statistical analysis

Data analysis was performed using SPSS 25.0 and R 4.4.2. Categorical variables were compared among the three groups using the Chi-square test. For continuous variables, one-way analysis of variance (ANOVA) was used when the data followed a normal distribution; otherwise, the Kruskal-Wallis H test was applied. Pairwise comparisons were conducted among the groups if the p-value was less than 0.05. To account for baseline differences across groups, propensity score matching (PSM) was performed using a caliper of 0.1 with a 1:1:1 matching ratio, utilizing the nearest neighbor matching method. Longitudinal changes between the groups were analyzed using linear mixed models (LMM). Bonferroni correction was applied to adjust for multiple comparisons. A p-value of less than 0.05 was considered statistically significant.

## Results

### Clinical characteristics

Before matching, there were imbalances in gender, age, and tumor size among the three groups. However, after performing 1:1:1 PSM, each group consisted of 106 patients, and the baseline characteristics of the three groups were well balanced, as shown in **Table 1**.

**Table 1:** Clinical characteristics of TOETVA, ETGUA, and COT before and after PSM.

Variable	Before matching (n=466)				After matching (n=318)			
	TOETVA (n=141)	ETGUA (n=168)	COT (n=157)	P	TOETVA (n=106)	ETGUA (n=106)	COT (n=106)	P
Gender				0.024				0.774
Male	27 (19.1)	21 (12.5)	38 (24.2)		19 (17.9)	17 (16.0)	21 (19.8)	
Female	114 (80.9)	147 (87.5)	119 (75.8)		87 (82.1)	89 (84.0)	85 (80.2)	
Age, years, median (IQR)	38.00 [34.00, 42.00]	40.00 [33.75, 44.00]	40.00 [35.00, 46.00]	0.034	38.50 [35.00, 42.00]	40.00 [33.00, 44.00]	39.00 [34.00, 44.00]	0.327
BMI, kg/m^2^, mean (SD)	20.29 ± 2.61	20.64 ± 2.83	20.74 ± 2.43	0.306	20.23 ± 2.72	20.66 ± 2.89	20.28 ± 2.32	0.443
Marital status				0.374				0.778
Married	88 (62.4)	104 (61.9)	87 (55.4)		67 (63.2)	62 (58.5)	64 (60.4)	
Single/Divorced/widowed	53 (37.6)	64 (38.1)	70 (44.6)		39 (36.8)	44 (41.5)	42 (39.6)	
Hypertension				0.392				0.705
Yes	17 (12.1)	20 (11.9)	26 (16.6)		16 (15.1)	15 (14.2)	12 (11.3)	
No	124 (87.9)	148 (88.1)	131 (83.4)		90 (84.9)	91 (85.8)	94 (88.7)	
Diabetes mellitus				0.101				0.564
Yes	12 (8.5)	15 (8.9)	24 (15.3)		9 (8.5)	9 (8.5)	13 (12.3)	
No	129 (91.5)	153 (91.1)	133 (84.7)		97 (91.5)	97 (91.5)	93 (87.7)	
Smoking				0.678				0.659
Yes	26 (18.4)	28 (16.7)	23 (14.6)		21 (19.8)	16 (15.1)	18 (17.0)	
No	115 (81.6)	140 (83.3)	134 (85.4)		85 (80.2)	90 (84.9)	88 (83.0)	
Drinking				0.159				0.476
Yes	20 (14.2)	38 (22.6)	32 (20.4)		17 (16.0)	21 (19.8)	24 (22.6)	
No	121 (85.8)	130 (77.4)	125 (79.6)		89 (84.0)	85 (80.2)	82 (77.4)	
ASA score				0.441				0.360
I	114 (80.9)	129 (76.8)	125 (79.6)		86 (81.1)	78 (73.6)	79 (74.5)	
II	23 (16.3)	30 (17.9)	21 (13.4)		18 (17.0)	21 (19.8)	19 (17.9)	
III	4 (2.8)	9 (5.4)	11 (7.0)		2 (1.9)	7 (6.6)	8 (7.5)	
Preoperative blood test								
WBC, ×10^9^/L, mean (SD)	7.2 ± 1.9	6.9 ± 1.7	7.0 ± 1.8	0.098	7.2 ± 1.9	6.9 ± 1.5	7.2 ± 1.8	0.351
CRP, mg/L, mean (SD)	5.0 ± 2.5	5.2 ± 2.5	4.8 ± 2.5	0.889	5.1 ± 2.5	5.2 ± 2.6	4.9 ± 2.7	0.677
IL-6, ng/L, mean (SD)	4.2 ± 1.8	4.0 ± 1.8	3.7 ± 1.8	0.821	4.2 ± 1.8	4.1 ± 1.8	3.9 ± 1.8	0.383
Size of tumor, mm, mean (SD)	9.6 ± 3.9	9.8 ± 4.0	10.8 ± 4.6	0.020	9.5 ± 3.5	9.4 ± 3.5	10.2 ± 3.9	0.235
Capsular invasion				0.176				0.490
Yes	26 (18.4)	21 (12.5)	31 (19.7)		16 (15.1)	15 (14.2)	21 (19.8)	
No	115 (81.6)	147 (87.5)	126 (80.3)		90 (84.9)	91 (85.8)	85 (80.2)	
Central neck dissection				0.126				0.239
Yes	104 (73.8)	129 (76.8)	105 (66.9)		78 (73.6)	75 (70.8)	67 (63.2)	
No	37 (26.2)	39 (23.2)	52 (33.1)		28 (26.4)	31 (29.2)	39 (36.8)	
N classification				0.577				0.239
N0	92 (65.2)	118 (70.2)	110 (70.1)		67 (63.2)	75 (70.8)	78 (73.6)	
N1	49 (34.8)	50 (29.8)	47 (29.9)		39 (36.8)	31 (29.2)	28 (26.4)	
TNM staging				–				–
I	141 (100)	168 (100)	157 (100)		106 (100)	106 (100)	106 (100)	

TOETVA, transoral endoscopic thyroidectomy vestibular approach; ETGUA, endoscopic thyroidectomy by gasless unilateral axillary approach; COT, conventional open thyroidectomy; ASA, American Society of Anesthesiologists; SD, standard deviation; IQR, interquartile range.

### Short-term outcomes

The completion rate for the baseline survey was 100%, while the rates for the postoperative surveys were 95.3% at 1 month, 91.5% at 3 months, 86.7% at 6 months, and 82.3% at 12 months.

TOETVA demonstrated the shortest incision length, although it required the longest operation time. ETGUA was associated with the highest volume of postoperative drainage, as well as the fewest retrieved lymph nodes.

No statistically significant differences were found among the three groups regarding blood loss, duration of drainage, number of metastatic lymph nodes, overall complications, inadvertent parathyroidectomy, postoperative hospital stay, or total hospital costs, as shown in **Table 2**.

**Table 2:** Short-term outcomes of TOETVA, ETGUA, and COT.

Parameters	TOETVA (A) (n=106)	ETGUA (B) (n=106)	COT (C) (n=106)	Overall p	P value of A VS. B	P value of A VS. C	P value of B VS. C
Length of incision, cm, mean (SD)	3.4 ± 0.4	8.3 ± 1.4	8.1 ± 1.3	< 0.001	< 0.001	< 0.001	0.563
Blood loss, ml, median (IQR)	19 (17, 22)	21 (16, 23)	20 (17, 24)	0.266	0.112	0.240	0.789
Operation time, min, median (IQR)	145 (132, 161)	125 (104, 133)	91 (81,101)	< 0.001	< 0.001	< 0.001	< 0.001
Amount of drainage, ml, median (IQR)	116 (105, 129)	152 (122, 165)	91 (82,100)	< 0.001	< 0.001	< 0.001	< 0.001
Duration of drainage, d, mean (SD)	3.0 ± 0.7	3.0 ± 0.8	3.1 ± 0.8	0.330	0.710	0.297	0.763
Retrieved LN, mean (SD)	6.8 ± 1.7	5.1 ± 1.5	7.1 ± 1.7	< 0.001	< 0.001	0.606	0.001
Metastatic LN, mean (SD)	0.9 ± 0.5	1.0 ± 0.4	1.1 ± 0.5	0.138	0.227	0.171	0.988
Complication							
Recurrent laryngeal nerve injury (temporary)	3	1	2	0.601	0.313	0.651	0.561
Recurrent laryngeal nerve injury (permanent)	0	0	0	-	–	–	–
Hypoparathyroidism (temporary)	17	13	21	0.326	0.431	0.474	0.134
Hypoparathyroidism (permanent)	0	1	2	0.364	0.316	0.155	0.561
Postoperative bleeding	3	0	1	0.170	0.081	0.313	0.316
Incision infection	1	1	2	0.776	1.000	0.561	0.561
Chyle leakage	0	0	0	–	–	–	–
Inadvertent parathyroidectomy	10	7	13	0.370	0.448	0.508	0.159
Postoperative hospital stay, d, mean (SD)	4.3 ± 0.8	4.5 ± 0.9	4.4 ± 0.8	0.135	0.113	0.629	0.523
Total hospital costs [RMB, median (IQR)]	16663.6 (14998.1, 22183.6)	16510.8 (14423.9, 21233.2)	16168.6 (14223.1, 18968.3)	0.159	0.355	0.070	0.238

TOETVA, transoral endoscopic thyroidectomy vestibular approach; ETGUA, endoscopic thyroidectomy by gasless unilateral axillary approach; COT, conventional open thyroidectomy; LN, lymph node; SD, standard deviation; IQR, interquartile range.

**Figure 3:**
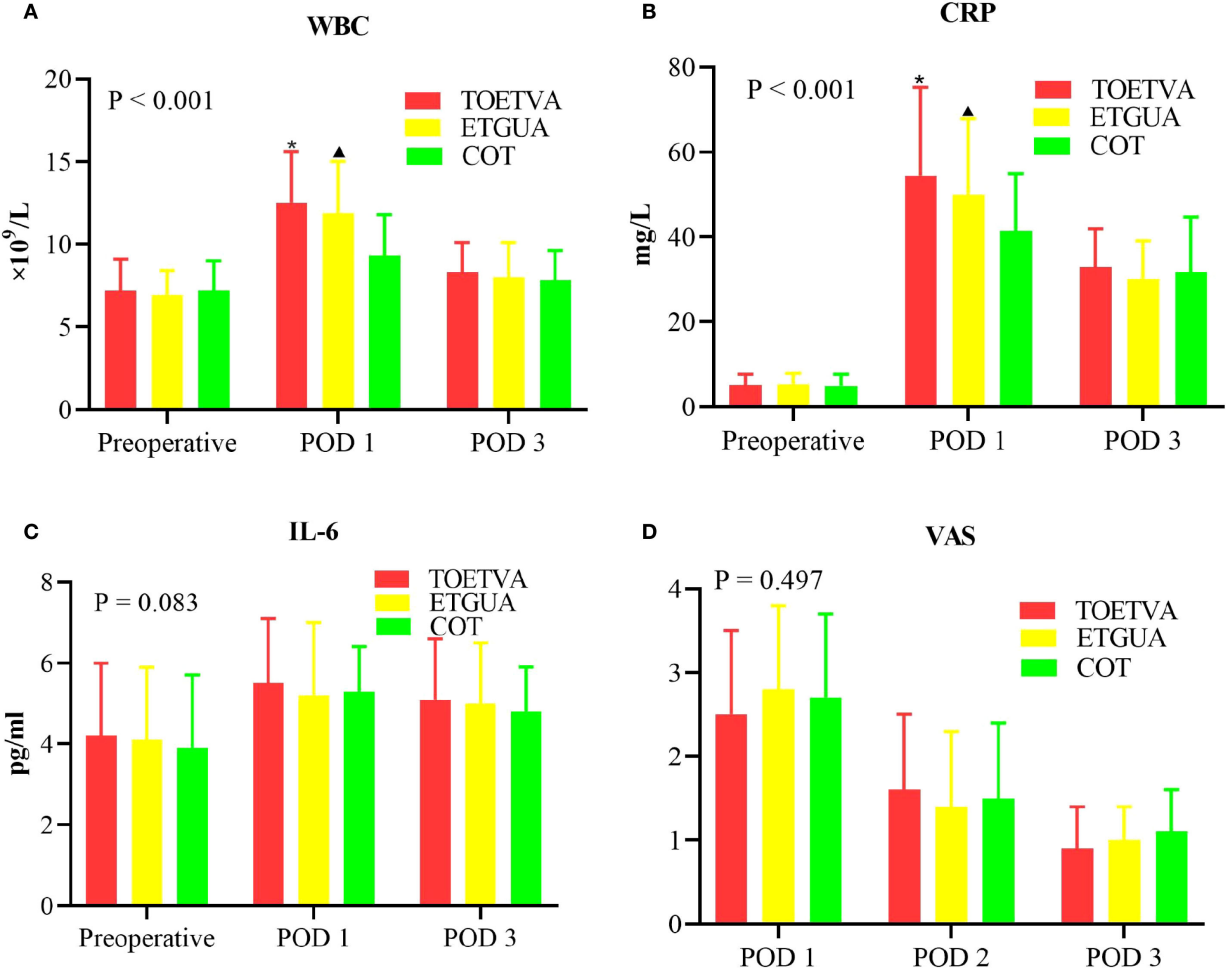
Postoperative inflammatory response and pain intensity. **(A)** WBC; **(B)** CRP; **(C)** IL-6; **(D)** VAS. ^*^indicates a statistically significant difference (p < 0.05) between TOETVA and COT; ^▴^indicates a statistically significant difference (p < 0.05) between ETGUA and COT.

On POD1, both TOETVA and ETGUA showed higher levels of WBC and CRP when compared to COT; however, by POD3, no significant differences were observed among the three groups. No differences in IL-6 or VAS scores were found among the groups postoperatively, as shown in **Figure 3**.

### Long-term outcomes

**Figure 4:**
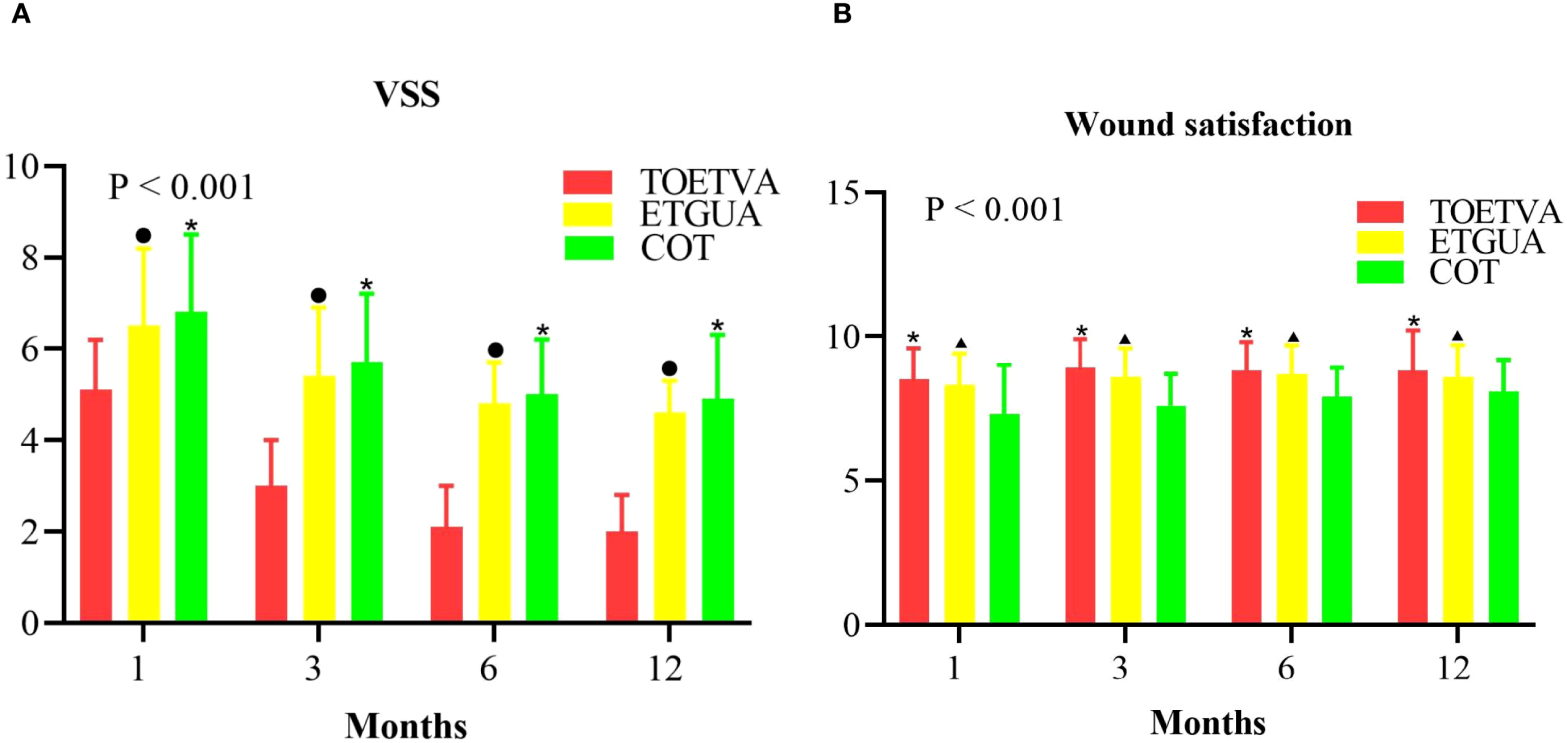
Comparison of postoperative scarring and wound satisfaction. **(A)** VSS; **(B)** Wound Satisfaction. ^•^indicates a statistically significant difference (p < 0.05) when comparing TOETVA and ETGUA; ^*^indicates a statistically significant difference (p < 0.05) between TOETVA and COT; ^▴^indicates a statistically significant difference (p < 0.05) when comparing ETGUA and COT.

TOETVA had the lowest VSS score, indicating the best scar appearance. However, no significant difference was observed between TOETVA and ETGUA in terms of wound satisfaction, with both approaches demonstrating higher satisfaction compared to COT, as shown in **Figure 4**.

**Figure 5:**
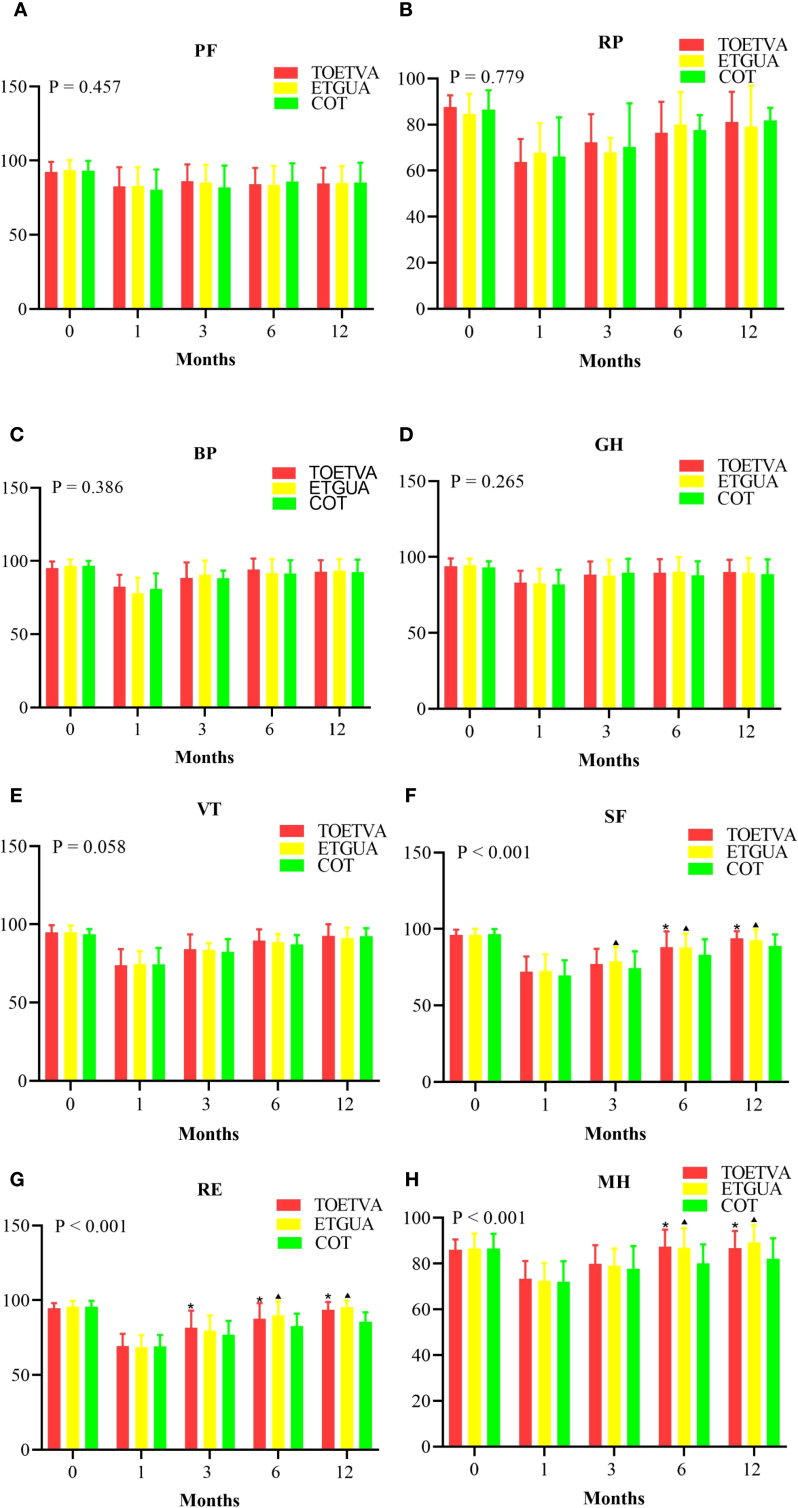
Comparison of Quality of Life Among the Three Patient Groups. **(A)** Physical functioning, **(B)** Role limitations due to physical problems, **(C)** Bodily pain, **(D)** General health perceptions, **(E)** Vitality, **(F)** Social functioning, **(G)** Role limitations due to emotional problems, **(H)** Mental health. ^*^indicates a statistically significant difference (p < 0.05) between TOETVA and COT; ^▴^indicates a statistically significant difference (p < 0.05) when comparing ETGUA and COT.

Regarding quality of life, there were no significant differences among the three groups in the dimensions of PF, RP, BP, GH, or VT. For SF, at 3 months postoperatively, ETGUA showed higher scores than COT. At 6 and 12 months, both TOETVA and ETGUA had higher SF scores compared to COT. In RE, at 3 months postoperatively, TOETVA showed higher scores than COT, and at 6 and 12 months, both TOETVA and ETGUA demonstrated higher scores compared to COT. For MH, both TOETVA and ETGUA showed higher scores than COT at 6 and 12 months postoperatively, as shown in **Figure 5**.

## Discussion

Laparoscopic thyroidectomy has become a widely adopted surgical technique for treating thyroid cancer, offering several advantages over traditional open surgery (22–25). However, within the field of laparoscopic surgery, multiple approaches exist, each with distinct benefits and limitations. At our institution, laparoscopic thyroid surgery was first extensively implemented in 2020, which facilitated the development of expertise and the standardization of procedures. This study focused on patients who underwent surgery starting in 2022, thereby minimizing the impact of the learning curve, which could otherwise introduce bias into outcome comparisons (26, 27).

In this study, TOETVA was associated with the longest operative time among the three techniques. This can be attributed to the confined working space of the oral vestibule and the complex surrounding anatomy, which demands enhanced surgical precision. The restricted space limits instrument maneuverability, often resulting in challenging dissection and prolonged operation time (28–30). While prolonged operative time is a known risk factor for complications due to extended anesthesia exposure (31), and while the transoral approach converts a naturally clean (Class I) incision into a potentially contaminated (Class II) one—theoretically increasing infection risk—no statistically significant differences were observed in overall complication rates across the groups. This discrepancy can be explained by several factors. First, all three surgical techniques are well-established at our institution, with standardized procedures implemented since 2020, thereby minimizing the impact of the learning curve and ensuring consistency and proficiency in surgical execution. Second, strict perioperative protocols were uniformly applied, including aseptic techniques, continuous intraoperative monitoring, and systematic postoperative care, helping to mitigate risks associated with prolonged surgery. Additionally, the minimally invasive nature of TOETVA may itself reduce infection risk (29, 32, 33). Moreover, although TOETVA was the only approach in which prophylactic antibiotics were administered throughout the perioperative period, the standardized surgical and antiseptic protocols (such as strict aseptic techniques and consistent postoperative wound care) applied across all groups likely also contributed to maintaining low and comparable infection rates.

ETGUA was associated with the highest volume of postoperative drainage; however, there were no differences in the duration of drainage among the three groups. This technique involves creating a surgical access pathway through a subcutaneous tunnel from the axillary incision to the neck. The operative space is relatively spacious, the pathway is longer, and the procedure requires significant traction and dissection of surrounding tissues. These factors contribute to an increased accumulation of interstitial fluid postoperatively, resulting in greater drainage. The axillary incision site, which involves relatively thick skin and subcutaneous tissue, is also rich in blood vessels and lymphatic channels. The surgical manipulation in this area can disrupt these vessels, leading to fluid accumulation in the surgical region (34, 35). Although elevated drainage volumes are often indicative of greater tissue trauma and may theoretically increase the risk of seroma or delayed wound healing (36), no statistically significant differences were observed in rates of postoperative bleeding, incision infection, or chyle leakage among the groups. This suggests that the increased drainage did not correlate with a higher incidence of adverse clinical events. Furthermore, although TOETVA and ETGUA were associated with elevated WBC and CRP levels on POD1—reflecting a more pronounced initial inflammatory response likely due to more extensive dissection and access route establishment (37)—these values normalized by POD3. No significant intergroup differences were found in WBC, CRP, or IL-6 levels at that time. The absence of differences in IL-6, a sensitive and short-half-life marker of surgical stress (38, 39), further indicates that the inflammatory response was transient and well-controlled.

Previous studies have shown that while performing contralateral thyroid lobectomy via a unilateral axillary approach presents certain technical challenges, experienced surgeons have demonstrated that total thyroidectomy and bilateral central compartment lymph node dissection through a single axillary incision are feasible (40–43). However, in this study, ETGUA was associated with the fewest retrieved lymph nodes (LN). The limited visibility of the contralateral central compartment lymph nodes, due to obstruction from the trachea, larynx, and cricoid cartilage, restricts access to these nodes, leading to fewer lymph nodes being dissected. Therefore, in cases where contralateral lymph node enlargement is suspected, careful consideration should be given to the selection of ETGUA as the surgical approach.

In this study, TOETVA demonstrated the shortest incision length and the lowest VSS score, indicating the best scar appearance among the three surgical approaches. The moist environment of the oral cavity and its rich vascular supply contribute to rapid wound healing and scar fading. TOETVA utilizes a transoral approach, which avoids visible external incisions, offering excellent cosmetic outcomes (18, 44, 45). Interestingly, no significant difference in wound satisfaction was observed between TOETVA and ETGUA, despite TOETVA having the lowest VSS score. The ETGUA incision is located within the axillary fold, providing a concealed surgical site that does not affect the aesthetic result (46, 47).

In this study, we also evaluated the total hospital costs associated with TOETVA, ETGUA, and COT, as cost is an important factor in the selection of surgical techniques. No statistically significant differences were observed in total hospital costs among the three groups. This suggests that, despite differences in operative time and technical complexity, the overall financial burden of these procedures is comparable, making them equally viable options from a cost perspective when considering other clinical and cosmetic outcomes.

In this study, overall, no significant differences were observed between the groups in the dimensions of PF, RP, BP, GH, or VT. These dimensions reflect general physical health and functioning, suggesting that, despite the differences in surgical techniques, all three approaches may yield similar physical outcomes in the early postoperative period. However, TOETVA and ETGUA demonstrated superior long-term benefits in the SF, RE, and MH domains. Both TOETVA and ETGUA contributed to reduced visible scarring, which enhanced patients’ overall mental health and body image. These improvements may, in turn, promote better emotional well-being and self-esteem (48–50).

This study has several limitations. First, it was conducted at a single center, which may limit the generalizability of the results to other institutions or patient populations. Second, this study did not assess long-term recurrence or survival outcomes due to the relatively short follow-up period. Future studies with extended follow-up are needed to evaluate the oncological efficacy of TOETVA, ETGUA, and COT. Finally, while robotic surgery has become increasingly prevalent in clinical practice (51), our institution initiated robotic surgery relatively recently, having performed only 10 cases at the time of the study. To minimize potential bias associated with the learning curve effect, robotic surgery was excluded from this analysis.

## Conclusions

Each surgical approach has its own advantages and limitations. TOETVA and ETGUA demonstrate superior outcomes in terms of incision satisfaction and specific dimensions of quality of life, providing significant benefits compared to COT. However, the selection of the surgical method should be individualized, considering both oncological effectiveness and the patient’s cosmetic and functional preferences.

## Data Availability

The original contributions presented in the study are included in the article/supplementary material. Further inquiries can be directed to the corresponding author.
